# Screening breast magnetic resonance imaging in women with hormone replacement therapy

**DOI:** 10.1186/s40001-018-0351-8

**Published:** 2018-10-20

**Authors:** Feng Zhang, Qingjing Feng, Zhiyong Zhang, Yanjun Hu, Zhifeng Zhang

**Affiliations:** 1 Department of Breast Surgery, Hangzhou Women’s Hospital, Hangzhou, 310008 China; 2 Department of Reproductive Endocrinology, Hangzhou Women’s Hospital, Kunpeng Road 369, Hangzhou, 310008 China; 3 Department of Breast Surgery, Yiwu Maternity and Child Care Hospital, Yiwu, 322000 China; 4 Reproductive Endocrinology, Women’s Hospital, School of Medicine, Zhejiang University, Hangzhou, 310006 China

**Keywords:** Hormone replacement therapy, Magnetic resonance imaging, Mammography, Breast cancer, Ductal carcinomas in situ

## Abstract

**Objective:**

The objective of this study was to compare the performance of screening mammography versus magnetic resonance imaging (MRI) in hormone replacement therapy (HRT) users.

**Methods:**

We performed a retrospective review of 4628 women who had mammography or breast MRI screening from the beginning of HRT use at three institutions from April 2005 to December 2015. Information of demographics, number of biopsies performed and pathologic outcomes were collected. Sensitivity, specificity, negative predictive value (NPV) and positive predictive value (PPV) of screening mammography and MRI were compared.

**Results:**

Totally 11,540 screening studies were collected, including 9580 mammography studies and 1960 MRI studies. Breast cancer was diagnosed in 26 patients. Of the 26 cancers, MRI detected 24 and mammography detected 15. For mammography, the sensitivity, specificity, PPV, and NPV were 57.7%, 99.1%, 14.6%, and 99.9%, respectively; for MRI, those values were 92%, 92.5%, 14.2%, and 99.9%, respectively. MRI screening was much more sensitive than mammography screening (*p* < 0.05, 92% vs 57.7%). There was no difference of specificity, PPV and NPV between two modalities.

**Conclusions:**

Our data showed that screening breast MRI may be a useful adjunct modality of mammography in HRT users.

## Introduction

The use of HRT has increased rapidly in China due to the expansion of the population of premature ovarian failure (POF) and more attention being paid to the side effects of menopause [1]. However, in many Western countries, influenced by the report from the first Women’s Health Initiative (WHI) randomized study, which concluded that the use of HRT was related to an increased risk of breast cancer, a cliff-like drop of HRT consumption was seen [2]. Subsequently, a decline in breast cancer incidence was also observed after the drop in HRT consumption in many countries [3]. Rudolph et al. proposed that modifications in gene polymorphisms related to solute transportation of mitochondria, immune response activation and transmembrane signaling are the possible explanation for the increased breast cancer risk [4].

Mammography is the primary screening modality to detect breast malignancy in the general population [5]. While for women who have the BRCA gene mutation, at least two first- or second-degree relatives with breast or ovarian cancer history and chest radiation history are recommended for mammography plus MRI screening annually [6]. Until now, scarce information has been available for the ideal screening modality in a non-high-risk or average high-risk population such as HRT users.

Although there are still disputes about the link between HRT and breast cancer, women taking HRT have always been afraid of increased breast cancer risk and would prefer annual breast cancer screening. Here, we study the performance of mammography and MRI screening in HRT users.

## Materials and methods

A retrospective review was completed on patients who had mammography or breast MRI screening after the beginning of HRT use at Hangzhou Women’s hospital, Zhejiang Provincial Women’s Hospital and Yiwu Maternity and Child Care Hospital from April 2005 to December 2015. The data of the screening studies, tissue biopsies, and pathologic findings were collected.

Comparisons of sensitivity, specificity, PPV, and NPV between mammography and MRI groups were performed using Fisher’s exact test and the methods described by Moscovitz [7].

## Results

Of 4628 HRT users, the mean age at the beginning of HRT use was 48 ± 4.1 years (range 43–56 years). The mean duration was 3.5 ± 1.8 years (range 1.2–6.5 years). All HRT users had mammography or breast MRI screening at least once after the beginning of HRT. There were 11,540 screening studies performed totally, including 9580 mammography studies and 1960 MRI studies.

There were 451 MRI studies and 2625 mammograms were assessed 1–2 by the Breast Imaging Reporting and Data System (BI-RADS) category. A total of 1364 MRI studies and 6867 mammograms got results of BI-RADS 3. There were 145 MRI studies and 88 mammograms were reported BI-RADS 4–5. Biopsy was made for all of those patients with BI-RADS 4–5. In total, there were 164 biopsies (3.5% of all patients), including 105 excisional biopsies and 59 core biopsies. There were 18 women assessed BI-RADS 1–3 by mammography while MRI were assessed BI-RADS 4–5. Pathology results from biopsy revealed eleven cancers (MRI true-positive) and seven benign results (MRI false-positive). There were six women assessed BI-RADS 1–3 by MRI while mammography were assessed BI-RADS 4–5. Pathology results from biopsy revealed two ductal carcinoma in situ (DCIS) and four benign results.

Of 4628 patients, 26 were diagnosed of breast malignancy. Of 26 cancers, 6 were diagnosed of DCIS and 20 were diagnosed of invasive breast cancers. Only one woman with interval cancer who had a negative mammography result at screening and touched a hard mass 6 months later that was diagnosed malignant by biopsy. Of 26 cancers, MRI detected 24 cancers and mammography detected 15 cancers. MRI missed only two cancers.

The sensitivity of MRI (92%) was significantly higher than mammography (57.7%) (*p* < 0.05). The comparison of specificity, NPV and PPV between mammography and MRI had no statistical significance (see Table 1).

**Table 1:** Diagnostic performance of screening MRI and mammography in HRT users

	MRI (95% CI)	Mammography I (95% CI)	*p*
Sensitivity	92 (85–96)	57.7 (52.4–65.4)	0.03
Specificity	92.5 (87.6–95.2)	99.1 (93.8–99.5)	0.26
PPV	14.2 (11.1–19.1)	14.6 (10.8–19.9)	0.94
NPV	99.9 (92.8–99.9)	99.9 (93.4–99.9)	0.96

*NPV* negative predictive value, *PPV* positive predictive value, *MRI* magnetic resonance imaging

## Discussion

Mammography was usually done as the primary modality for breast cancer screening, while for those at a high risk, MRI took priority over mammography [6, 8]. Since scarce information is available for the ideal screening modality for average-risk patients like HRT users, the performance of mammography and MRI screening in women HRT users were compared in our study.

Although some disputes exist based on three large epidemiology studies, it was concluded that the risk of breast cancer increased with exposure to combination HRT [9, 10]. Lee et al. [11] proposed that the odds ratio (OR) could increase by 7.6% for each year of combination HRT use linearly. And at the tenth year, the HR could reach between 1.5 and 2.5. The relationship of HRT and breast cancer risk was established mainly for the phenomenon that breast cancer incidence decreased after the decline of HRT use, which was observed in many countries [12].

Studies demonstrated that no effect of HRT use was observed in younger women (< 50 years) and the HR of breast cancer following HRT use increased with age at first use [13]. More than half of breast cancer patients in Western countries were older than 50 or postmenopausal when diagnosed, while most patients in China were under 50 years old or premenopausal. The mean age of this cohort at the beginning of HRT use was 48 ± 4.1 years (range 43–56 years). That means there may be some differences in the population between China and Western countries. The results of Korean and Japanese studies indicated women who received HRT had no increase of breast cancer risk [14, 15]. Hou et al. [16] also suggested that different populations might have various breast cancer risks from HRT. They further concluded that Asian women with low BMI and dense breasts were associated with greater risk than other populations.

Although HRT use may decrease sensitivity and specificity of mammography, it still is the first choice for breast cancer screening for those patients [17, 18]. Until now, there has been no reporting on MRI screening in HRT users. In our study, MRI had significantly higher sensitivity than mammography (*p* < 0.05, 92% vs. 57.7%). There were 11 cancers (42%) found by MRI alone. Mammography detected only two additional cancers. MRI had a higher false-positive rate and was less specific than mammography [19]. In our study, the negative–positive patients in MRI screening were 145 and in mammography were 88. The specificities were 92.5% and 99.1% of MRI and mammography, respectively. There was no statistical significance of specificity, PPV, and NPV between two modalities.

PPV was low in both mammography and MRI modalities in our study. The possible explanation may be that Chinese women have smaller-size breast and denser breast tissue, who were younger than western women when menopause occurred. The mean age of the cohort of this study at the beginning of HRT use was 48 ± 4.1 years (range 43–56 years). Chinese women had high rate of fibroadenomas and fibrocystic proliferation in their breasts, which raised the suspicion of malignancy.

There were six diagnoses of DCIS in our study. Four were detected by MRI and only two were detected by mammography. According to prior studies, MRI was superior to mammography in detecting DCIS [20]. Our study confirmed this conclusion. Two DCIS patients presented micro-calcifications in mammography, which could be detected more reliably by mammography than MRI [21].

The debate about the application of MRI in screening mainly focuses on its low positive biopsy, increased call-back, greater cost than mammography, and poor patient compliance [22, 23]. MRI detection needs a lot of time, requires the injection of intravenous gadolinium, and many patients experience claustrophobia in the MRI device. In our cohort, only about 600 patients received MRI as a screening modality, about one-eighth of all HRT users. There were 164 biopsies performed (3.5% of all patients), including 105 excisional biopsies and 59 core biopsies. Benign pathological results included fibroadenomas, fibrocystic proliferation, atypia and complex sclerosing lesions.

The main limitation of our study was its retrospective design. The patients had random time intervals between mammography and MRI screening rounds. They also had random HRT use durations. It is hard to calculate the real duration of drug usage, which also made it difficult to tell whether or not the occurrence of breast cancer was due to HRT use. Many patients may choose combined screening modality such as ultrasound, mammography, and MRI. And some may choose 1, 2, or all 3, which limits the ability to compare mammography and MRI.

In conclusion, our study confirmed that breast MRI in HRT users was more sensitive than mammography. Because MRI was seldom taken as a screening modality alone, it could be a useful adjunctive modality with mammography in screening. When physicians recommend a screening mode to HRT users, they should pay attention to the benefit and deficiency of MRI screening, especially for women who are younger at menopause and have denser breast tissue.

## Abbreviations

MRI: magnetic resonance imaging
HRT: hormone replacement therapy
NPV: negative predictive value
PPV: positive predictive value
POF: premature ovarian failure
WHI: Women’s Health Initiative
BI-RADS: breast imaging reporting and data system
DCIS: ductal carcinoma in situ
OR: odds ratio
